# PKM1调控小细胞肺癌自噬与神经内分泌标志物表达

**DOI:** 10.3779/j.issn.1009-3419.2024.102.33

**Published:** 2024-09-20

**Authors:** Chenchen TANG, Yulong JIN, Peiyan ZHAO, Lin TIAN, Hui LI, Changliang YANG, Rui ZHONG, Jingjing LIU, Lixia MA, Ying CHENG

**Affiliations:** ^1^130012 长春，吉林省肿瘤医院生物样本库; ^1^Biobank, Jilin Cancer Hospital, Changchun 130012, China; ^2^130012 长春，吉林省肿瘤医院肿瘤转化医学实验室; ^2^Medical Oncology Translational Research Lab, Jilin Cancer Hospital, Changchun 130012, China; ^3^130012 长春，吉林省肿瘤医院肿瘤内科; ^3^Department of Medical Oncology, Jilin Cancer Hospital, Changchun 130012, China

**Keywords:** 肺肿瘤, PKM1, 自噬, 神经内分泌, 药物敏感性, Lung neoplasms, PKM1, Autophagy, Neuroendocrin, Drug sensitivity

## Abstract

**背景与目的:**

小细胞肺癌（small cell lung cancer, SCLC）恶性程度高，异质性强，被称为难治性肿瘤。免疫治疗改变了广泛期SCLC（extensive-disease SCLC, ED-SCLC）的治疗格局，但受益人群有限，因此，寻找新的治疗措施是目前SCLC亟待解决的临床问题。SCLC具有高度活跃的糖酵解代谢特征，而丙酮酸激酶M1（pyruvate kinase M1, PKM1）是糖酵解途径中重要的限速酶PK的同工酶之一。研究表明PKM1与自噬及药物敏感性有关，但PKM1如何调控SCLC药物敏感性及其作用机制尚不清楚。本研究旨在探讨PKM1在SCLC中的生物学功能，包括PKM1对SCLC的增殖、迁移、自噬、药物敏感性及神经内分泌（neuroendocrine, NE）相关标志物表达的影响。

**方法:**

应用Western blot检测SCLC细胞中PKM1的表达水平；通过慢病毒稳定转染构建PKM1基因过表达的SCLC细胞株，MTT法检测细胞增殖能力及药物敏感性，Transwell实验测定细胞迁移能力，流式细胞术检测细胞自噬水平，Western blot检测NE相关蛋白的表达水平。

**结果:**

不同的SCLC细胞系PKM1表达具有差异性，H1092中PKM1表达较低（P<0.01）。与对照组相比，PKM1过表达的H1092细胞虽然增殖水平无明显差异，但迁移能力增高（P<0.001），药物敏感性降低，并且自噬水平受到抑制（P<0.001）。此外过表达PKM1可上调非神经内分泌（non-neuroendocrine, non-NE）相关蛋白表达（P<0.01），降低NE相关蛋白表达（P<0.01）。

**结论:**

PKM1在SCLC细胞系中具有差异表达，PKM1高表达不影响SCLC的细胞增殖，但影响其迁移。PKM1可能通过抑制自噬，调节NE标志物的表达，从而影响药物敏感性，研究结果为探索PKM1在SCLC中的作用提供了理论依据。

## Abstract (en)

**Background and objective:**

Small cell lung cancer (SCLC) is known as recalcitrant cancer with high malignancy and heterogeneity. Immunotherapy has changed the treatment pattern of extensive-disease SCLC (ED-SCLC), but the beneficiary population is limited. Therefore, exploring new therapeutic strategies is an urgent clinical problem to be solved for SCLC. SCLC is characterized by highly active glycolytic metabolism and pyruvate kinase M1 (PKM1) is one of the isozymes of PK, an important rate-limiting enzyme in glycolysis pathway. Previous studies have shown that PKM1 is related to autophagy and drug sensitivity, however, how PKM1 regulates drug sensitivity in SCLC and its mechanism remain unclear. The aim of this study was to investigate the biological functions of PKM1 in SCLC, including its effects on proliferation, migration, autophagy, drug sensitivity, and expression of neuroendocrine (NE)-related markers in SCLC.

**Methods:**

Western blot was used to detect the expression level of PKM1 in SCLC cells. PKM1 gene-overexpressed SCLC cell lines were constructed by stable lentivirus transfection. Proliferation of cells and drug sensitivity were detected by MTT, and migration ability of cells was determined by Transwell. The level of autophagy was detected by flow cytometry. Western blot was used to determine the expression levels of NE-related proteins.

**Results:**

PKM1 was differentially expressed among various SCLC cell lines, and was lower in H1092 cells (P<0.01). Compared with the control group, there was no significant difference in proliferation level of PKM1 overexpressing H1092 cell, but the migration ability was significantly increased (P<0.001), the drug sensitivity was reduced, and the level of autophagy was inhibited (P<0.001). Additionally, overexpression of PKM1 could upregulate the expression of non-neuroendocrine (non-NE)-related proteins (P<0.01) and decrease the expression of NE-related proteins (P<0.01).

**Conclusion:**

PKM1 was differentially expressed in SCLC cell lines, and high expression of PKM1 did not affect the proliferation, but affected the migration of SCLC cells. PKM1 might affect drug sensitivity by inhibiting autophagy and regulating the expression of NE markers. These results provide a theoretical basis for exploring the role of PKM1 in SCLC.

肺癌是目前全球死亡率最高的恶性肿瘤，小细胞肺癌（small cell lung cancer, SCLC）具有高度侵袭性，约占所有肺癌患者的15%^[1,2]^。尽管大多数SCLC患者对初始化疗较为敏感，但很快会复发耐药，因此，广泛期SCLC（extensive-disease SCLC, ED-SCLC）患者的5年生存率不足2%^[3]^。目前免疫治疗改变了ED-SCLC的治疗格局，但受益人群有限，因此，探索SCLC新的潜在治疗靶点，寻找新的治疗措施是目前SCLC亟待解决的临床问题。

SCLC由不同的细胞亚群构成，包括神经内分泌（neuroendocrine, NE）细胞和非神经内分泌（non-NE, non-NE）细胞，不同的亚型均具有特异性的生物标志物。SYP是突触小泡的主要膜蛋白，是NE细胞最可靠的标志物之一^[4]^。而HES1是碱性螺旋-环-螺旋（basic helix-loop-helix, bHLH）蛋白家族的一员，在non-NE细胞中表达，在NE细胞中不表达^[5]^。NE表型与non-NE表型之间可互相转化，表型转化可能是导致SCLC发生耐药及患者低生存的重要机制^[6]^。

恶性肿瘤周围环境相对缺氧，肿瘤细胞中的糖酵解途径会异常激活，提供增殖所需能量。丙酮酸激酶M（pyruvate kinase M, PKM）是糖酵解反应中的重要限速酶，催化磷酸烯醇式丙酮酸转化为烯醇式丙酮酸，进而产生能量。PKM有两种异构体，即PKM1与PKM2，两者由来自同个基因转录本的选择性剪接产生^[7]^，PKM1缺少10号外显子，PKM2缺少9号外显子。虽然只有1个外显子的差别，但它们的功能却截然不同。PKM2已被证实在多种癌症中过表达，并可以促进肿瘤细胞的增殖和转移^[8]^，但是目前缺乏对肿瘤中PKM1功能的深入研究。自噬是一种细胞降解自身细胞器和大分子物质的过程。首先在胞质中形成一个双膜结构的自噬体，其包裹细胞物质与溶酶体融合，导致细胞物质降解^[9]^。自噬与癌症密切相关，但其在癌症中的作用复杂，表现出肿瘤抑制或肿瘤促进作用。在肿瘤抑制方面，自噬可通过抑制应激和活性氧来起到抑制肿瘤的作用^[10]^。肿瘤促进方面，自噬通过多种癌基因激活和抑癌基因失活来支持晚期肿瘤的生长和代谢，调节自噬可用于癌症治疗^[11]^。

PKM1可以促进肿瘤细胞的生长，在NE肿瘤表现尤为明显^[12]^，提示PKM1可能在SCLC细胞生长中具有重要作用。本研究通过探索SCLC中PKM1的表达特征，并且通过调控PKM1表达探究其对SCLC细胞增殖、迁移、药物敏感性、自噬能力以及NE、non-NE标志物表达的影响，以揭示PKM1在SCLC中的功能。

## 1 资料与方法

### 1.1 实验仪器及试剂

KHB-ST-360酶标仪购自上海科华生物工程有限公司；FACScanto流式细胞仪购自美国BD公司；GBOXF3凝胶成像系统购自美国Syngene公司；S1000梯队PCR仪购自美国Bio-Rad公司；DMI 3000显微镜购自德国Leica；DMEM培养基（货号：C3110-0500）、RPMI-1640培养基（货号：C3010-0500）、胎牛血清（货号：C04001-500）购自上海VivaCell生物技术有限公司；Lipofectamine 2000（货号：11668-019）购自美国Invitrogen生命技术公司；MTT（货号：88417）、DMSO（货号：34943）购自美国Sigma-Aldrich贸易有限公司；SDS-PAGE蛋白上样缓冲液（货号：P0015L）购自上海碧云天生物技术有限公司；蛋白预制胶（货号：ET15420LGel）购自南京ACE生物科技有限公司；PKM1、SYP、HES1、LC3抗体购自美国Cell Signaling生物公司；PKM2、GAPDH抗体、山羊抗兔免疫球蛋白G（immunoglobulin G, IgG）购自沈阳万类生物科技有限公司；质粒小提试剂盒（货号：DP103-02）购自中国TIANGEN生物技术公司；逆转录试剂盒（货号：FSQ-101）、SYBR Green Master Mix试剂盒（货号：D7260）购自日本TOYOBO生物科技有限公司；pMD2G、psPAX2、PKM（65885-1）-shRNA及对照质粒、实时定量聚合酶链式反应（real-time quantitative polymerase chain reaction, RT-qPCR）引物与内参购自上海吉凯基因医学科技有限公司。

### 1.2 实验方法

#### 1.2.1 细胞培养

NCI-H69（H69）、NCI-H2227（H2227）、NCI-H1092（H1092）细胞培养于RPMI-1640培养基，NCI-H446（H446）、HEK293T细胞培养于DMEM培养基，培养基中均含有10%的胎牛血清和1%的青霉素/链霉素，在37 ^o^C、5% CO_2_、饱和湿度的培养箱中培养。

#### 1.2.2 慢病毒稳定转染细胞系建立

使用Lipofectamine 2000和慢病毒包装质粒pMD2G和psPAX2在HEK293T细胞中进行PKM1过表达质粒及对照质粒转染，连续3 d收集细胞上清中病毒并使用PEG8000浓缩病毒，使用病毒感染H1092细胞。通过嘌呤霉素筛选阳性细胞。嘌呤霉素从0.2至1.0 µg/mL浓度梯度递增，最终得到稳定过表达PKM1的H1092细胞株（H1092PKM1+细胞）及稳定转染空载质粒的H1092-control细胞株。

#### 1.2.3 siRNA转染

转染前24 h，将H2227细胞以5×10^5^个/孔的密度接种到6孔板中。细胞达到70%的密度，使用脂质体Lipofectamine 2000将siRNAs（PKM1Ri）转染到细胞中，4 h后更换完全RPMI-1640培养基。转染后2 d，获得H2227PKM1-细胞，收集细胞进行下一步实验。

#### 1.2.4 细胞增殖检测 H1092

PKM1+及H1092-control以4000个/孔的密度接种于96孔板中，24 h后每孔添加20 µL MTT试剂（5 mg/mL），37 °C孵育4 h。每孔加入150 µL DMSO，摇床中震荡10 min后，使用酶标仪在570 nm处测量吸光度（optical density, OD）值。计算细胞增殖抑制率公式：（OD_H1092-control组_值-OD_H1092 PKM1+组_值）/OD_H1092-control组_值×100%。

#### 1.2.5 Western blot

细胞弃去培养基，加入1 mL PBS清洗1遍，加入适量RIPA裂解缓冲液（含1 mmol/L PMSF）。4 °C、12,000 rpm离心15 min，分离上清液用于后续分析。用BSA法测定总蛋白，每孔30 µg蛋白加5 µL 5×蛋白上样缓冲液进行10%聚丙烯酰胺凝胶电泳。电泳160 V待蛋白质分离后，加入转膜液电泳100 V、90 min，将蛋白质转移到聚偏氟乙烯微孔膜上，并在室温下用含0.1% Tween-20的TBS（TBST）洗涤3次，用5%脱脂牛奶阻断1 h。膜在TBST缓冲液中洗涤3次，并加入一抗PKM1（1:500）、LC3（1:1000）、SYP（1:1000）、HES1（1:500）与GAPDH（1:1000）孵育。放入4 ^o^C冰箱中在摇床上过夜。一抗孵育后，膜用TBST洗涤3次，用辣根过氧化物酶标记的山羊抗兔IgG室温下孵育2 h。膜用TBST洗涤3次，加入ECL曝光试剂，使用凝胶成像系统检测蛋白信号，并通过Quantity One软件测量灰度值。

#### 1.2.6 Transwell迁移实验

H1092PKM1+及H1092-control在无血清培养基中培养24 h。使用24孔板，Transwell小室使用8.0 μm孔膜。上腔室中加入200 μL无血清培养基，下腔室中加入500 μL完全培养基作为趋化剂。将H1092-control及H1092PKM1+细胞以1×10^5^个/孔分别接种在上腔中。37 °C孵育48 h，用棉签去除残留在膜上表面的细胞，膜下表面的细胞为迁移细胞。用4%多聚甲醛固定，再用0.1%结晶紫溶液染色后，用倒置荧光显微镜拍摄。

#### 1.2.7 MTT法测定细胞活力

将H1092PKM1+及H1092-control细胞接种在96孔板中（5×10^3^个/孔）孵育48 h，加入MTT，37 °C孵育4 h，加入DMSO溶解MTT。490 nm处测量吸光度。建立西奥罗尼、尼拉帕利、鲁比卡丁的浓度-反应曲线，将不同浓度的西奥罗尼（0、0.5、1、2、4、8、16、24 µmol/L）、尼拉帕利（0、0.5、1、2、4、8、16、24 µmol/L）、鲁比卡丁（0、0.005、0.01、0.02、0.04、0.08、0.16、0.32 nmol/L）对细胞进行处理。为了检测自噬抑制剂3-MA对H1092PKM1+细胞的活性抑制作用，在H1092PKM1+细胞中加入3-MA（0、1、2、3、4、5 nmol/L）孵育48 h，使用GraphPad Prism 8.2统计软件计算半数抑制浓度（half inhibitory concentration, IC_50_）。在H1092PKM1+细胞中加入IC_50_值浓度的3-MA孵育24 h，并收集细胞进行下一步实验。

#### 1.2.8 细胞自噬的检测

选取对数生长期的H1092PKM1+细胞及H1092-control细胞，弃去培养液，并用预冷的PBS洗涤细胞3次。然后消化细胞并收集到流式管中，以1500 rpm离心5 min。再次用PBS洗涤细胞，重悬并在相同条件下离心3次，使用1×Binding buffer悬起细胞，单丹磺酰尸胺（monodansylcadaverin, MDC）染色。37 °C染色30 min，PBS清洗细胞2次。流式细胞仪检测染色细胞^[13]^。

#### 1.2.9 RT-qPCR实验

使用RNA迷你试剂盒提取各组细胞的总RNA，OD_260 nm_/OD_280 nm_>1.95为RNA质量合格。使用逆转录试剂盒进行逆转录。使用SYBR Green Master Mix试剂盒对样品进行RT-qPCR分析。设置循环如下：95 °C预变性3 min，在预变性之后，95 °C变性30 s，使DNA双链分离，然后将温度设置为57 °C，进行30 s的退火，使引物结合到单链DNA上，最后，将温度调至72 °C，进行延伸30 s，合成新的DNA链，共进行40个循环。

计算公式：用2^-ΔΔCT^方法计算目的基因的相对定量，即ΔCT值=CT_目的基因_值-CT_GAPDH_值， 以GAPDH为对照； ΔΔCT=ΔCT_PKM1过表达组_值-ΔCT_对照组_值， 以对照组为对照。每个实验至少重复3次。引物序列： PKM1正义链-CACACTGGACTAGTGGATCCCGCCACCATGTCGAAGCCCCATAG-3’， 反义链CACTTAAGCTCGGCACAGGAACAACACGCATGG-3'。

### 1.3 统计学分析

所有数据均采用GraphPad Prism 8.2统计软件进行分析。结果报告表示为Mean±SD。多组间比较采用方差分析，两组间差异比较应用t检验，以P<0.05为差异有统计学意义。

## 2 结果

### 2.1 不同SCLC细胞类型中PKM1、LC3及SYP表达水平不同

为了探究PKM1在SCLC中的作用，我们使用Western blot检测SCLC细胞系H1092、H69、H446、H2227细胞中PKM1蛋白的表达情况。结果显示，H1092细胞中PKM1蛋白表达水平较低（图1A、1B）。随后我们检测了H1092、H69、H446、H2227中自噬标志物LC3及SYP的蛋白表达情况，发现H1092细胞LC3的表达水平与其他细胞之间无明显差异（P>0.05）。与H446、H2227相比，H1092与H69的NE标志物SYP表达水平较高（P<0.0001）（图1C、1D）。

**图1:**
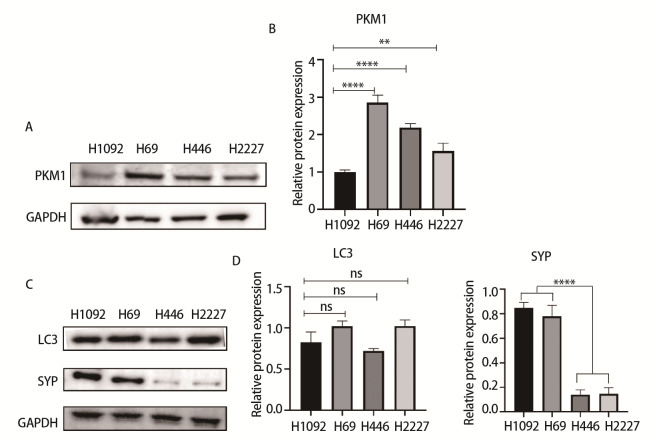
不同SCLC细胞中PKM1、自噬和NE相关的蛋白表达情况。A：Western blot检测H1092、H69、H446、H2227细胞中PKM1表达；B：H1092、H69、H446、H2227细胞中PKM1蛋白表达比例的统计图；C：Western blot检测H1092、H69、H446、H2227细胞中自噬标志物LC3和NE标志物SYP的表达情况；D：H1092、H69、H446、H2227细胞中LC3、SYP蛋白表达比例的统计图。**P<0.01；****P<0.0001。

### 2.2 建立过表达PKM1的SCLC细胞模型

为验证稳转细胞系H1092PKM1+细胞的PKM1表达情况，利用RT-qPCR及Western blot分别检测H1092PKM1+细胞中PKM1的mRNA和蛋白表达水平。结果发现，与H1092-control相比，H1092PKM1+细胞中PKM1蛋白的表达水平升高（P<0.05）（图2A、2B），mRNA表达水平升高（P<0.0001）（图2C），证明成功获得过表达PKM1的H1092细胞。

**图2:**
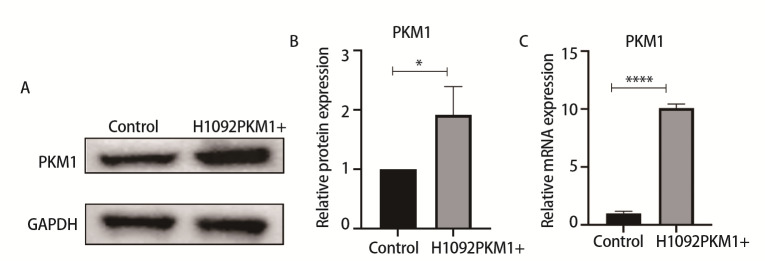
在H1092细胞中PKM1呈过表达。A：Western blot鉴定H1092PKM1+细胞中PKM1蛋白水平；B：Western blot鉴定H1092PKM1+细胞PKM1蛋白水平的半定量分析图；C：RT-qPCR鉴定H1092PKM1+细胞PKM1 mRNA水平。*P<0.05；****P<0.0001。

### 2.3 过表达PKM1不影响SCLC细胞增殖，但可促进细胞迁移，降低细胞对药物的敏感性

为了探究过表达PKM1对SCLC生物学功能的影响，我们使用MTT法检测H1092PKM1+细胞和H1092-control细胞培养3 d后的细胞活性，结果显示，H1092PKM1+细胞与H1092-control细胞的活性无统计学差异（P>0.05）（图3A）。采用Transwell实验检测H1092PKM1+细胞和H1092-control细胞的迁移能力，结果显示，与对照组相比，H1092PKM1+细胞的迁移能力增高（P<0.001）（图3B、3C）。

**图3:**
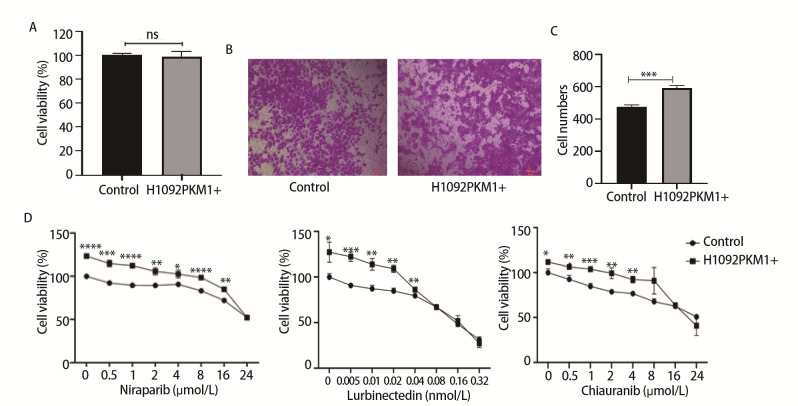
过表达PKM1对H1092细胞增殖、迁移及药物敏感性的影响。A：H1092对照组与H1092PKM1+组细胞的增殖率对比；B：H1092对照组与H1092PKM1+组细胞的迁移能力（×100）；C：H1092对照组与H1092PKM1+组细胞的迁移率；D：H1092对照组与H1092PKM1+组细胞中加入尼拉帕利（0-24 µmol/L）、鲁比卡丁（0-0.32 nmol/L）、西奥罗尼（0-24 µmol/L），48 h后MTT法检测细胞活性。*P<0.05；**P<0.01；***P<0.001；****P<0.0001。

为了进一步探究PKM1对SCLC细胞药物敏感性的影响，加入不同浓度的鲁比卡丁、西奥罗尼和尼拉帕利，MTT法检测药物处理后H1092PKM1+细胞及H1092-control细胞的活性。结果显示，H1092PKM1+细胞活性均高于H1092-control细胞（P<0.05）（图3D），说明PKM1过表达降低了H1092细胞对化疗药物和部分靶向药物的敏感性。

### 2.4 过表达PKM1抑制SCLC细胞自噬

自噬与药物敏感性密切相关，为进一步探究PKM1影响药物敏感性的机制，我们采用流式细胞术检测H1092PKM1+细胞与H1092-control细胞中发生自噬的细胞比例，结果显示，H1092PKM1+细胞中发生自噬的细胞比例低于H1092-control细胞（P<0.001）（图4A、4B）。随后采用Western blot检测了H1092PKM1+组与H1092-control组自噬标志物LC3的表达，结果显示，与H1092-control组对比，H1092PKM1+细胞的LC3表达水平降低（P<0.001）（图4C）。上述研究结果说明PKM1能够抑制SCLC细胞发生自噬。为了进一步探究PKM1与自噬的关系，在H1092PKM1+组中加入自噬抑制剂3-MA，结果显示，随着3-MA的剂量增加，H1092PKM1+的细胞活性降低，呈剂量依赖性，其IC_50_值为3.048 nmol/L（P<0.0001）（图4D）。在H1092PKM1+中加入3-MA后，结果显示，与H1092-control组相比，H1092PKM1+组的LC3的表达水平降低（P<0.001）（图4E、4F）。

**图4:**
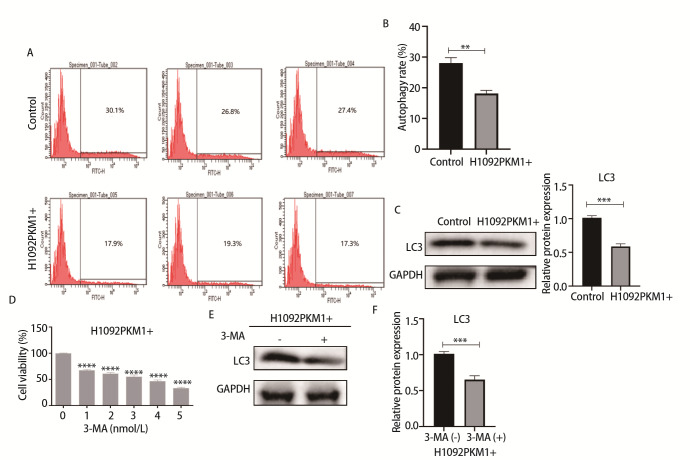
过表达PKM1对H1092细胞自噬的影响。A：H1092-control组与H1092PKM1+组经MDC染色后，通过流式细胞术分析自噬情况；B：流式细胞术检测H1092-control组与H1092PKM1+组中发生自噬细胞比例的统计图；C：Western blot鉴定H1092PKM1+细胞LC3蛋白水平及分析统计图；D：H1092PKM1+组细胞中加入自噬抑制剂3-MA（0-5 nmol/L），48 h后MTT法检测细胞活性；E：Western blot检测H1092PKM1+细胞中加入自噬抑制剂3-MA前后的LC3蛋白表达水平；F：H1092PKM1+细胞中加入自噬抑制剂3-MA前后的LC3蛋白表达水平的统计图。***P<0.001；****P<0.0001。

### 2.5 过表达PKM1调控SCLC细胞NE标志物SYP及non-NE标志物HES1

SCLC的NE亚型与药物敏感性具有相关性。我们采用Western blot检测了H1092PKM1+细胞及H1092-control细胞NE相关蛋白SYP以及non-NE相关蛋白HES1的表达。结果发现，与H1092-control组相比，H1092PKM1+细胞HES1表达水平升高（P<0.01），NE标志物SYP蛋白表达水平降低（P<0.01）（图5A）。为了进一步探究PKM1、自噬与NE之间的关系，在H1092PKM1+中加入自噬抑制剂3-MA。结果显示，加入3-MA后，与H1092-control组相比，H1092PKM1+组的SYP的表达水平升高（P<0.05），HES1表达水平降低（P<0.001）（图5B）。

**图5:**
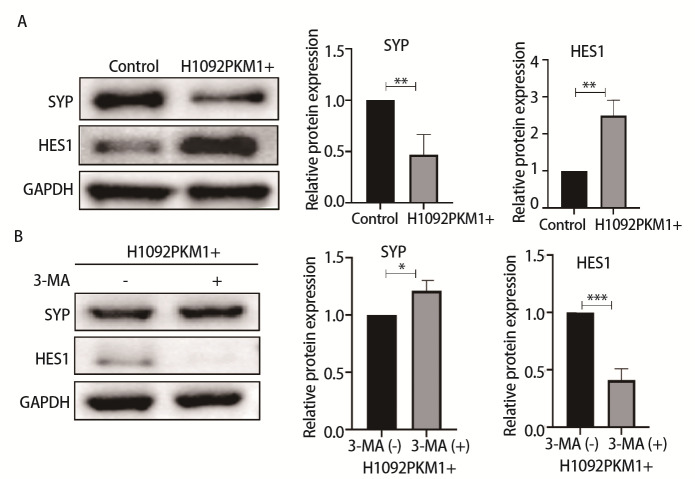
对比加入自噬抑制剂前后过表达PKM1对H1092细胞NE和non-NE标志物表达水平的影响。A：过表达PKM1对H1092细胞SYP和HES1表达水平的影响及分析统计图；B：加入自噬抑制剂3-MA后SYP及HES1的表达水平及分析统计图。*P<0.05；**P<0.01；***P<0.001。

### 2.6 在PKM1表达水平高的SCLC细胞中敲低PKM1，NE标志物无改变

在高表达PKM1的H2227细胞中敲低PKM1（H2227PKM1-细胞）。利用Western blot检测PKM1蛋白表达，结果显示，与H2227-control细胞相比，H2227PKM1-细胞中PKM1的表达水平降低（P<0.01），同时NE标志物SYP的表达水平无明显变化（P>0.05）（图6A、6B）。

**图6:**
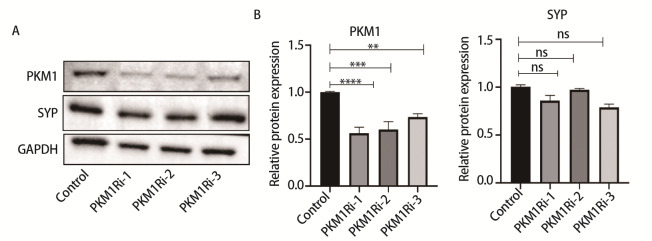
在H2227细胞中敲低PKM1对NE标志物的影响。A：Western blot检测H2227-control组与H2227PKM1-组中PKM1及NE标志物SYP的表达水平；B：H2227-control组与H2227PKM1-组中PKM1及NE标志物SYP表达水平的统计分析图。**P<0.01；***P<0.001；****P<0.0001。

## 3 讨论

SCLC约占所有肺癌患者的15%，但仍然是最致命的恶性肿瘤之一。其恶性程度高，以早期发生转移扩散为特征，且极易发生复发耐药^[14,15]^。虽然免疫治疗改变了SCLC的治疗格局，但由于SCLC的肿瘤异质性及耐药等原因，导致受益人群仍有限^[16]^，因此，探索SCLC新的潜在治疗靶点至关重要。PKM1是糖酵解反应中的重要限速酶之一，通过调节糖酵解途径，在多种癌症中发挥作用，包括结直肠癌及肺腺癌等。有研究^[17]^显示，敲低PKM1促进了结直肠癌细胞的增殖，并上调了与DNA复制和细胞周期过渡相关的细胞周期调节因子的表达。在结直肠癌中PKM1的高表达伴随着细胞耐药性降低^[18]^，此外有研究^[19]^表明在肺腺癌细胞中敲除PKM1，可促进自噬导致细胞死亡，由此可见PKM1有可能是肺癌中的一个潜在靶点。本研究发现，过表达PKM1可以抑制SCLC的自噬，non-NE标志物表达显著升高，NE标志物表达显著降低，且药物敏感性降低，表示靶向PKM1可能改变SCLC的恶性生物学行为。

肿瘤细胞中瓦博格效应（Warburg effect）的一种重要代谢特征。这种效应是指肿瘤细胞在有氧条件下依然依赖糖酵解来供给能量。PKM1是糖酵解反应中的重要限速酶。有研究^[12]^显示，大多数癌症细胞均低表达PKM1，本研究在4种SCLC细胞中检测PKM1的表达，发现除H1092细胞外，其余3种SCLC细胞均高表达PKM1。我们在H1092细胞中过表达PKM1，发现过表达PKM1对其增殖能力没有明显影响。因此我们猜测PKM1可能通过其他途径影响SCLC细胞。

Kim等^[18]^使用药物处理结肠癌细胞，发现随着细胞对药物敏感性的降低，细胞中的PKM1表达增高，这与本研究的结果一致。PKM1过表达后SCLC细胞的药物敏感性降低，揭示了PKM1对SCLC药物敏感性的调控作用。细胞自噬被证明与肿瘤细胞的药物敏感性密切相关，但自噬和耐药性之间的关系具有两面性，自噬既可以诱导肿瘤细胞耐药以启动自我保护机制，也能诱导细胞死亡，增加肿瘤对药物的敏感性^[20,21]^。在Xiang等^[22]^的研究中，乳腺癌自噬活性降低后，对紫杉醇的敏感性降低，但是目前也有联合自噬抑制剂组合的新治疗策略，抑制自噬后，使细胞对药物的敏感性增高^[21]^，上述研究均证明了自噬与药物敏感性的不一致性。本研究的结果显示，在SCLC细胞中，PKM1过表达后，细胞自噬水平降低，药物敏感性降低，这可能与肿瘤类型不同有关。

SCLC是一种高度异质性肿瘤，包含NE和non-NE表型，两种表型可相互转化^[23]^。Li等^[24]^的研究发现，在食管鳞状细胞、胆囊癌和甲状腺乳头状癌中，PKM1的异常表达与表型转化呈正相关。本研究团队前期的研究表明SCLC表型转化与细胞NE特征、安罗替尼、西奥罗尼和依维莫司等药物敏感性以及药物靶点表达相关^[25]^。本研究在SCLC中过表达PKM1后，自噬水平降低，non-NE标志物表达显著升高，NE标志物表达显著降低，提示PKM1可能参与了NE/non-NE表型改变。随后我们在过表达PKM1的基础上加入了自噬抑制剂，发现NE标志物表达升高，non-NE标志物表达降低，说明PKM1可能通过自噬调控SCLC的NE/non-NE表型转化，导致细胞亚型的改变，进而影响SCLC药物敏感性。有研究^[26]^显示，在NE前列腺癌（prostate cancer, PCa）细胞中，抑制自噬，可以导致NE PCa细胞的NE标志物水平降低以及细胞存活率降低，表明自噬激活是NE PCa细胞存活和NE表型改变所必需的，这与本研究发现的PKM1调控SCLC细胞NE表型的结果一致。这提示PKM1可能是调控SCLC药物敏感性的较有价值的潜在靶点。

但本研究也存在局限性，我们并未在动物体内或临床样本中进行验证。未来，我们将从细胞及临床样本等多个层面对PKM1通过调控自噬参与SCLC表型及药物敏感性改变的分子机制进行深入探索，为克服SCLC耐药提供可能的靶点及治疗策略。
